# Quantification and reporting of vitamin D concentrations measured in human milk by LC–MS/MS

**DOI:** 10.3389/fnut.2023.1229445

**Published:** 2023-11-16

**Authors:** Kerry S. Jones, Sarah R. Meadows, Albert Koulman

**Affiliations:** Nutritional Biomarker Laboratory, MRC Epidemiology Unit, University of Cambridge, Cambridge, United Kingdom

**Keywords:** infant feeding, vitamin D, 25-hydroxyvitamin D, breast milk, mass spectrometry

## Abstract

Vitamin D is essential for optimal bone health, and vitamin D deficiency has been associated with an increased risk of adverse pregnancy, growth and developmental outcomes. In early life, and in the absence of endogenous vitamin D production from UVB light, infants are reliant on vitamin D stores established *in utero* and the vitamin D supply from human milk (HM). However, comprehensive data on vitamin D in HM is lacking. Thus, in this review we explore the application of liquid-chromatography tandem mass spectrometry (LC–MS/MS) to the assessment of vitamin D in HM. We discuss the challenges of extracting and measuring multiple vitamin D metabolites from HM including the frequent requirement for a large sample volume, and inappropriate poor sensitivity. Shortcomings in the reporting of experimental procedures and data analysis further hinder advances in the field. Data collated from all studies that have applied LC–MS/MS reveal that, in general, cholecalciferol concentration is greater and more variable than 25-hydroxyvitamin D concentration, and that the vitamin D content of HM is low and less than the currently recommended dietary requirement of infants, although maternal supplementation can increase the vitamin D content of HM. Improvements in analytical methods and their validation and larger, more representative studies are required to better characterize HM milk vitamin D metabolite concentrations and their relationship with maternal status. These data are essential to understand relationships with infant health and to inform public health policies around vitamin D fortification and supplementation.

## 1. Introduction

Vitamin D is necessary for optimal bone health across the life course (1). In neonates and infants, hypocalcaemia caused by vitamin D deficiency may have a number of health effects with consequences for growth and development (2). Inadequate vitamin D intake, concurrent with insufficient calcium intake, can manifest as rickets with its associated health effects (2). The risk of rickets can be reduced with sufficient vitamin D intake (3).

Although vitamin D status has been associated with many other health outcomes (4), meta-analyses of observational data and vitamin D supplementation trials in pregnancy, post-partum, or infancy, provide limited evidence for a beneficial effect of vitamin D in relation to growth or immunological functions (5–8). Nevertheless, adequate vitamin D status is essential to prevent vitamin D deficiency and both known and unconfirmed vitamin D-related health effects.

Neonates and young infants rely on vitamin D from stores accumulated *in utero* through the placenta, from UVB-induced cutaneous vitamin D synthesis and dietary intake. Umbilical cord venous blood 25-hydroxyvitamin D (25(OH)D) concentration correlates with, but is typically 60 to 80% of the maternal 25(OH)D venous blood concentration (9). As a consequence, in any mother-infant pair, infants are at greater risk of vitamin D deficiency. Even if maternal vitamin D status is adequate, the circulating half-life of 25(OH)D of 2–3 weeks (10) means that vitamin D stores obtained *in utero* may not sustain the vitamin D status of the infant beyond 8 weeks (2). In many areas of the world, public health guidance is that infants under 6 months of age should not be exposed to direct UVB light (11, 12) and therefore they are reliant primarily on oral vitamin D supply from human milk (HM) or formula milk. HM is considered the optimum food for infants, providing all the nutrients necessary for growth and development (13, 14).

Despite global recommendations that infants under 6 months should be exclusively breastfed (15), information on HM micronutrient content is limited – not only in the scale of the data available but also its quality due to differences in collection methods, inadequate measurement and method validation, and non-standardized reporting (16). For vitamin D, there are limited data on HM concentrations and how these relate to infant requirements. Indeed, in a recent review of literature to determine the status of evidence of milk micronutrient content, the authors found only one suitable study for inclusion on vitamin D (17). A comprehensive 2018 review of evidence used to inform recommended intakes in USA/Canada, UK, Europe and from WHO, found that no vitamin D milk concentration data were used, partly because what data were available reported low concentrations (18).

Analytical methods applied to HM vitamin D measurement have included immunoassays (19) and chromatography-based methods (20–22) or combinations of both approaches with ligand binding assays following purification by different techniques including lipid extraction, saponification, and silica and chromatographic purification (23, 24). Sensitive and specific liquid chromatography tandem mass spectrometry (LC–MS/MS) methods are considered the ‘gold standard’ for serum/plasma measurement of vitamin D metabolites. While the advent and growth of use of LC–MS/MS has led to major developments and progress in vitamin D measurement (25), relatively few studies have applied this technique to the analysis of HM vitamin D.

Reviews have considered from an analytical chemistry perspective the extraction and measurement of vitamin D from different tissues (26), including milk (27), using different analytical approaches and using chromatographic techniques and LC-MS/MS (28). Another review also considered some of the data on the anti-rachitic activity of HM (29). Recently a systematic review and meta-analysis reviewed vitamin D content of HM, but did not provide a detailed breakdown of vitamin D concentrations from individual studies (30). In order to interpret the published data it is also necessary to be conscious of some of the strengths and weaknesses of the analytical methods. Therefore, in this review, we discuss the assessment of vitamin D metabolites content in HM using LC–MS/MS. We collate all published vitamin D milk concentration data obtained with LC–MS/MS, and review these in the context of the analytical methods. In addition, we review the suitability of the described assays and the reporting of the methods and data. The aim is to improve our understanding of these areas in order to help facilitate use of HM vitamin D data in policy decisions and for future research.

### 1.1. Overview

Using PubMed keywords “(“vitamin D”) AND (“human milk” or “breast milk” or “breastmilk” [All Fields])” restricted to humans and from the year of first publication of LC–MS/MS (1987), 306 hits were returned. Only manuscripts with HM concentrations measured by LC–MS/MS were reviewed. The search was conducted in February 2023. An additional manuscript was identified that described a method for vitamin D in HM (31), resulting in 17 original manuscripts. One manuscript focused on sulphated vitamin D metabolites in serum and HM and was not considered further (32). In addition, a study that reported serum 24,25(OH)_2_D_3_ concentration and HM vitamin D_3_ after supplementation (33) was not included since the HM data was the same as that published previously (34). Consequently, 15 manuscripts were reviewed in detail. One additional manuscript was later identified and included in the review (35). Three manuscripts described methods and provided data on a small number of convenience or commercial HM samples (31, 36, 37). Four reports provided only brief method details and data from randomized controlled trials of maternal vitamin D supplementation (34, 38, 39). The remaining manuscripts gave either full or partial descriptions of an analytical method that was applied to observational studies across different population groups at different stages postpartum and in different countries with sample sizes ranging from a few to around 100 individuals.

In the sections below, we review the generation of HM vitamin D concentrations, from the initial sample collection, through analysis and reporting.

## 2. Pre-analytical factors: sample collection protocols and macronutrient content

Where reported, methods and protocols for the collection of HM are summarized in Supplementary Table S1. The included level of detail is highly variable. HM collections were performed with manual expression or with a breast pump and included complete expression, either foremilk, hindmilk or midstream (stored separately or combined), or were collected at unspecified points. Samples were collected at different times of the day and time postpartum. Collected volumes are rarely described.

Macronutrient composition of HM is highly variable across lactation, during the day and even during a feed (40) and may affect vitamin D content. Kamao et al. observed a significant positive correlation between 25(OH)D_3_ milk concentration and milk fat content (41). Another study showed significantly higher concentrations of 25(OH)D_3_ in hindmilk compared to foremilk but similar concentrations of vitamin D_3_, results that were not considered to support the association between fat content and vitamin D content (42). Season was shown to affect the milk concentration of vitamin D_3_ and, to a lesser extent, 25(OH)D_3_ (42, 43) consistent with other non-LC–MS/MS studies (44) and with known seasonal variation in vitamin D status due to the availability of UVB light necessary for the cutaneous synthesis of vitamin D_3_. Evidence from the studies reviewed here suggests no effect of lactation stage and time since delivery (38, 41, 42) on milk vitamin D content, although one study was cross-sectional (41). Differences between colostrum and mature milk have not been specifically investigated, nor the relationship with milk volume.

## 3. Analytical factors

### 3.1. Extraction methods

Extraction methods for vitamin D metabolites in HM for analysis by LC–MS/MS have included combinations of some or all of saponification, protein precipitation (PP), liquid–liquid extraction (LLE) with different solvents, solid phase extraction (SPE) and semi-preparative HPLC (Table 1) and were recently reviewed (28).

**Table 1:** Summary of methods for the analysis of vitamin D in human milk using liquid chromatography tandem mass spectrometry.^1,2^

Author	Method reference (if different)	Method summary	Sample volume (mL)	LOD (nmol/L)	LOQ (nmol/L)	Intra-assay (%CV)	Inter-assay (%CV)	Recovery (%)
Kamao, 2007(41)		Saponification, LLE (hexane:ethyl acetate), semi-preparative HPLC and DMEQ-TAD derivatisation	10	2.7–5.3	NR	NR	4–12	82–110%
Oberhelman, 2013 (34)	Based on method for serum LC–MS/MS method 25(OH)D (45)	LLE (isopropyl alcohol) and separation with online extraction	NR	2.5	18.2	8	6.1	104%
Amukele 2013 (46)	Hoofnagle 2010 (47)	NR	NR	0.25	NR	NR	NR	100%
Ferreiro-Vera 2013 (37)		Centrifugation to separate milk serum and lipids. Online automated SPE.	0.2	0.05–0.39	0.12–1.3	0.8–2.3	1.1–4.6	85–100%
Jan Mohamed, 2014 (48)	Based on serum LC–MS/MS method for 25(OH)D (49) (citing (50))	NR	NR	2	NR	NR	NR	NR
Gomes, 2015 (31)		PP (acetonitrile), LLE (hexane:dicholormethane), PTAD derivatisation	4	0.00027–0.00047	0.00095–0.00117	4.8–13.2	NR	88–106%
Gomes, 2016 (51)	Gomes 2015 (31)	NR	4	0.0012–0.0017	0.0042–0.0058	3.9–7.9	NR	82–109
Vio Streym 2016 (42)		Saponification and LLE (heptane)	NR	0.2	NR	4.3–8.3	NR	NR
Wall, 2016 (38)	Competitive binding assay method, Hollis 1993 (52)	Saponification, LLE (dichloromethane), SPE, semi-preparative HPLC	5–10	0.025	NR	≤12	≤12	NR
Stoutjesdijk, 2017 (53)	Extraction method based on blood spots for GCMS, Corso 2002 (54); LC–MS/MS Kamao 2007 (41)	Saponification, LLE (hexane) and DMEQ-TAD derivitisation	0.5	NR	0.1–0.2	10	<15	NR
Stoutjesdijk, 2019 (39)	Corso 2002 (54); Kamao 2007 (41)	Saponification, LLE (hexane) and DMEQ-TAD derivitisation	NR	NR	0.1–0.2	<10	<15	NR
Dawodu, 2019 (35)	Wall (38)	Solvent extraction, semi-preparative HPLC	NR	NR	NR	<12% (38)	<12% (38)	NR
Gjerde, 2020 (55)		Automated PP (ZnSO4 and methanol) and SPE	0.2	0.5–2.1	1.7–7.0	4–16	5–17	72–103%
Wang 2020 (56)		PP (acetonitrile), LLE (methanol and hexane)	1	NR	NR	NR	NR	NR
Oberson 2020 (36)		PP (ethanol) and LLE (hexane:ethyl acetate). PTAD derivitisation [supercritical fluid chromatography-MS]	1	NR	0.05	7.1–37.3	10.7–32.7	100–112%
Tsugawa 2021 (43)	Kamao 2007 (41)	Saponification, LLE (hexane:ethyl acetate), SPE and DMEQ-TAD derivitisation	10	0.001	NR	5.3–43.8	2.0–9.7	NR

^1^Analytical performance statistics are summarized for 25(OH)D_2_, 25(OH)D_3_, vitamin D_2_ and vitamin D_3_.^2^All units have been converted to nmol/L for ease of comparison. To convert nmol/L to ng/mL divide by 2.4 for 25(OH)D_2_, 2.5 for 25(OH)D_3_ and vitamin D_2_ and 2.6 for vitamin D_3_.

%CV, % coefficient of variation; GCMS, gas chromatography – mass spectrometry; HPLC, high performance liquid chromatography; NR, not reported; LOD, limit of detection; LOQ, limit of quantitation; LLE, liquid/liquid extraction; PP, protein precipitation; SPE, solid phase extraction.

Saponification and PP are used to disassociate vitamin D metabolites from proteins, including vitamin D binding protein (DBP). In addition, saponification is used to break up fat globules that may contain vitamin D metabolites. In direct comparisons, chemical PP led to 1.5 to 2-fold better recovery than saponification (31, 36), possibly due to degradation and cross reactions of compounds during saponification. Different solvents have been used for PP including acetonitrile, methanol and ethanol. Similarly, a wide range of solvents, alone in or combination were used for LLE, partly reflecting optimisation of procedures for different vitamin D metabolites and their different polarities. These have included hexane (39, 48, 53), heptane (42), isopropanol (34), methanol (55), dichloromethane (38) or a mix of hexane:ethyl acetate (9:1) (36, 41, 43).

### 3.2. Sensitivity limits and derivatisation

Descriptions of sensitivity are not always included nor consistently applied and it is therefore difficult to compare sensitivity between methods. Some manuscripts specify a limit of detection (LOD), limit of quantitation (LOQ), or both, but do not always describe how the limits were defined (Table 1). Limits may be presented as units measured in the original sample volume, others in the solvent extract and others in the amount injected onto the chromatography column.

Not all studies described how results below the detection limit were handled during data analysis, a relevant factor that may influence study conclusions. Two studies reviewed here reported using assigned values of either half the detection limit (43) or LOD/sqrt(2) (42) and other studies used assigned values. Others included all the data, but either only reported a range (39) or used the values and reported that their inclusion had no effect on the study conclusions (53). One study did not report results less than the LOQ (31).

Analytical sensitivity can be improved with derivatisation, the chemical alteration of a molecule to improve mass spectrometric detection by increasing ionization efficiency and the molecular weight of the compound to reduce chemical noise. Four manuscripts (39, 41, 43, 53) from two groups reported using DMEQ-TAD (4-[2-(6,7-dimethoxy-4-methyl-3-oxo-quinoxalin-2-yl)ethyl]-1,2,4-triazole-3,5-dione) and two others, PTAD (4-phenyl-1,2,4-triazole-3,5-dione) (31, 36) (Table 1). As expected, analytical sensitivity was higher with the use of derivatisation; LOQs ranged from 0.001 to 0.1 nmol/L (31, 36, 39, 53) and LODs from 0.0003 to 0.001 nmol/L (31, 43) with derivatisation. In studies that did not use derivatisation, reported LOQ ranged from 4.3 nmol/L to 18.2 nmol/L (34, 55) and LOD from 0.025 to 2.5 nmol/L (38, 42, 48). Ferrerio-Vera et al., reported lower LOD and LOQ for underivatised samples than other studies (0.125 nmol/L for 25(OH)D_3_ and vitamin D_3_) (37).

### 3.3. Choice of vitamin D metabolites and reporting

Despite the knowledge that both vitamin D and 25(OH)D may contribute to anti-rachitic activity (ARA) in HM, not all studies have taken advantage of the specificity of LC–MS/MS methods to measure both metabolites; three studies only measured 25(OH)D (48, 55, 56). The concentrations of vitamin D_2_ forms are considerably lower than vitamin D_3_ forms (31, 36, 38, 39, 43, 53) and often less than the detection limit. However, Gomes et al. reported similar concentrations of vitamin D_2_ and vitamin D_3_ in HM, although mean 25(OH)D_3_ concentration was double that of 25(OH)D_2_ (51).

Studies have presented vitamin D concentrations as either ng/ml, pmol/L or nmol/L. Some studies reported ARA either together with concentration data or alone (35) to describe HM vitamin D content which provides the ability to combine concentrations of 25(OH)D and vitamin D. The ARA assigns higher biopotency to 25(OH)D, such that 1 IU/L ARA equals 25 pg/mL vitamin D and 5 pg/mL 25(OH)D (53). A general issue in some vitamin D literature is the unquantified use of the term ‘vitamin D’ as short-hand for 25(OH)D (e.g., (46)), which may lead to confusion where individual or both forms are of interest.

## 4. Concentration data

An overview of reported 25(OH)D and vitamin D concentrations in HM is presented from observational (Table 2) and randomized controlled trials (Table 3). In observational studies from women from different continents and countries, including New Zealand, Denmark, USA, Japan, Malaysia, Vietnam, Taiwan and Tanzania, HM 25(OH)D_3_ concentrations typically ranged between 0.1 to 1.4 nmol/L and vitamin D_3_ concentrations between 0.1 to 7.8 nmol/L.

**Table 2:** Human milk vitamin D concentration data – observational studies.^1^

Author/ Country	Sub-groups/time point	*n*	Result format	25(OH)D_2_ (nmol/L)	25(OHD)_3_ (nmol/L)	Vitamin D_2_ (nmol/L)	Vitamin D_3_ (nmol/L)
Kamao 2007 (41); Japan	All 0–10 days pp 11–30 days pp 31–90 days pp 91–180 days pp 181–270 days pp	82 8 43 18 8 5	Mean (range) Mean (SD)	0.007 (0–0.029) 0.007 (0.000) 0.017 (0.007) 0.001 (0.005) 0.007 (0.007) 0.007 (0.002)	0.203 (0.058–0.430) 0.203 (0.093) 0.180 (0.118) 0.210 (0.085) 0.170 (0.093) 0.183 (0.103)	0.187 (0–3.120) 0.187 (0.374) 0.310 (0.182) 0.175 (0.478) 0.158 (0.202) 0.434 (0.238)	0.229 (0.026–2.902) 0.195 (0.120) 0.268 (0.439) 0.205 (0.146) 0.195 (0.205) 0.091 (0.042)
Amukele 2013 (46); Malawi	Delivery 2 months pp	21 21	NR	0 0	0 0	NM	NM
Ferreiro-Vera 2013 (37); Spain		5	Mean (SD)	ND	ND	23.0 (5.90)	16.0 (1.73)
Jan Mohamed, 2014 (48); Malaysia	1–14 days pp 2 months pp 6 months pp 12 months pp	101 90 69 49	Mean (95% CI)	NR	1.26 (1.09, 1.42) 1.18 (1.09, 1.27) 1.01 (0.99, 1.04) 1.16 (1.02, 1.31)	NM	NM
Gomes, 2015 (31); Australia	n/a	NR	Mean	0.0092	0.013	0.002	0.016
Gomes, 2016 (51); Australia	Pre-pasteurization Post-pasteurization	16 16	Mean (SD)	0.0016 (0.0002) 0.0013 (0.0001)	0.0033 (0.0022) 0.0031 (0.0022)	0.0041 (0.0005) 0.0034 (0.0004)	0.0042 (0.0042) 0.0038 (0.0024)
Vio Streym, 2016 (42); Denmark	Fore milk 2 weeks pp 4 months pp 9 months pp Hind milk 2 weeks pp 4 months pp 9 months pp	106 [48]^2^ 90 [50] 48^3^ [34] 101 [64] 84 [68] 41 [32]	Median - all data [median excluding vitamin D3 results <0.2 nmol/L]^2^	Present in 5 samples overall	0.9 0.8 0.9 1.3 1.4 1.2	Present in 1 sample overall	0.1 [0.5]^2^ 0.3 [0.7] 0.4 [0.9] 0.3 [0.7] 0.8 [1.4] 0.6 [1.0]
Stoutjesdijk 2017 (53); Various countries	The Netherlands Curacao Vietnam-Halong Bay Vietnam-Phu Tho Vietnam-Tien Giang Vietnam-Ho Chi Minh City Vietnam-Hanoi Malaysia-Kuala Lumpur Tanzania-Ukerewe Tanzania-Maasai	9 10 20 22 20 18 21 20 21 20	Median (range)		0.50 (ND – 0.63) 0.23 (0.06–0.55) 0.52 (0.27–0.76) 0.24 (ND – 0.73) 0.43 (0.15–1.08) 0.43 (0.22–0.71) 0.38 (0.11–0.89) 0.11 (ND – 0.55) 0.32 (ND – 1.03) 0.72 (0.42–1.13)		0.25 (0.02–0.41) 0.80 (0.02–4.50) 0.56 (0.09–3.99) 0.21 (0.05–1.52) 1.75 (0.15–10.9) 0.88 (0.15–3.86) 0.21 (0.04–3.01) 0.15 (0.05–0.68) 2.66 (0.52–13.4) 1.93 (0.20–7.78)
Wang, 2020 (56); Taiwan	Atopic dermatitis infants Control	45 45	Mean (SD)	NM	4.3 (0.75) 9.9 (1.6)	NM	NM
Gjerde, 2020 (55); Norway	6 weeks pp 6 months pp	137	Mean (SD)	12.8 (6.7) 12.7 (7.1)	60.1 (24.8) 50.0 (23.4)	NM	NM
Oberson 2021 (36); USA	Commercially-sourced sample	7	Range	<0.05	0.32–0.65	0.09–0.16	0.17–7.83
Tsugawa, 2021 (43); Japan	1989 2016–17	72 90	Mean (SD)	0.006 (0.014) 0.018 (0.035)	0.322 (0.312) 0.261 (0.130)	0.073 (0.120) 0.045 (0.138)	0.252 (0.477) 0.161 (0.174)

^1^All units have been converted to nmol/L for ease of comparison. To convert nmol/L to ng/mL divide by 2.4 for 25(OH)D_2_, 2.5 for 25(OH)D_3_ and vitamin D_2_ and 2.6 for vitamin D_3_. To convert to ‘anti-rachitic activity’ (ARA) 25 pg/mL vitamin D_3_ is equal to 1 IU/L and 5 pg/mL of 25(OH)D_3_ is equal to 1 IU/L (53).

^2^Due to the high number of samples with vitamin D_3_ less than the limit of quantitation (0.2 nmol/L), median vitamin D_3_ data are presented (a) assuming 0.14 nmol/L for samples < 0.2 nmol/L and (b) excluding values < 0.2 nmol/L, in square brackets.

^3^*n* = 48 for vitamin D_3_, *n* = 47 for 25(OH)D_3_.

ND, not detected; NM, not measured; NR, not reported; pp, postpartum.

**Table 3:** Human milk vitamin D concentration data – randomized controlled trials.^1^

Author/ Country	Intervention	Groups	*n*	Result format	25(OH)D_2_, nmol/L	25(OHD)_3_, nmol/L	Vitamin D_2_, nmol/L	Vitamin D_3_, nmol/L	ARA (IU/L)
Oberhelman, 2013 (USA)	1–6 months postpartum, 28 day intervention with either daily dosing (5,000 IU/d) or a single bolus dose (150,000 IU)	5,000 IU/d Day 0 Day 1 Day 3 Day 7 Day 14 Day 28 150,000 IU (bolus) Day 0 Day 1 Day 3 Day 7 Day 14 Day 28	20 20	Mean (SD)	NM	ND	NM	< 18.2 < 18.2 20.8 (9.6) 18.7 (12.5) 22.4 (14) 20.0 (9.6) < 18.2103.2 (42.1) 64.0 (23.1) 29.1 (12.2) < 18.2 < 18.2	NR
Wall, 2016 (New Zealand)	Maternal supplementation trial (1,000 or 2,000 IU vitamin D3/d between 27 weeks gestation and delivery)	2 weeks pp Placebo 1,000 IU/d 2,000 IU/d 2 months pp Placebo 1,000 IU/d 2,000 IU/d	22 27 22 19 15 14	Median (range)	< 0.026 < 0.026 < 0.026 < 0.026 < 0.026 < 0.026	0.55 (0.22–1.77) 0.55 (0.11–3.33) 0.60 (0.20–1.60) 0.55 (0.20–1.18) 0.55 (0.20–1.35) 0.43 (0.07–1.55)	< 0.026 < 0.026 < 0.026 < 0.026 < 0.026 < 0.026	0.21 (0.03–7.72) 0.44 (0.03–3.20) 0.94 (0.16–6.55) 0.23 (0.08–1.69) 0.26 (0.44–1.14) 1.12 (0.10–6.60)	NR
Dawodu, 2019 (Qatar)	Maternal supplementation with 600 IU or 6,000 IU vitamin D3/d from 1 month to 6 months pp	600 IU 4 weeks pp 4 months pp 6 months pp 6,000 IU 4 weeks pp 4 months pp 6 months pp	90 60 41 92 68 54	Median (range)	NR	NR	NR	NR	8.1 (8.1, 379.9) 12.1 (8.1, 70.8) 14.3 (8.1, 203.1) 8.1 (8.1, 532.0) 185.9 (8.1, 64.0) 143.7 (8.1, 852.5)
Stoutjesdijk, 2019 (The Netherlands)	400–3,100 IU/d provided between 20 weeks gestation and 4 weeks pp	10 (400 IU) μg/d 35 (1,400 IU) μg/d 60 (2,400 IU) μg/d 85 (3,400 IU) μg/d	7 8 11 7	Median (range)	ND ND ND ND	0.34 (0.22–0.49) 0.54 (0.35–0.85) 0.61 (0.42–12.45) 0.57 (0.46–0.74)	ND ND 0.11 (ND – 0.20) ND (ND – 0.26)	0.4 (ND – 1.6) 1.5 (1.0–6.2) 5.6 (0.7–14.3) 7.5 (3.7–16.0)	NR

^1^All units have been converted to nmol/L for ease of comparison. To convert nmol/L to ng/mL divide by 2.4 for 25(OH)D_2_, 2.5 for 25(OH)D_3_ and vitamin D_2_ and 2.6 for vitamin D_3_. To convert to ‘anti-rachitic activity’ (ARA) 25 pg/mL vitamin D_3_ is equal to 1 IU/L and 5 pg/mL of 25(OH)D_3_ is equal to 1 IU/L (53). For supplementation dose, 400 IU is equal to 10 μg. ND, not detected; NM, not measured; NR, not reported; pp, postpartum.

Vitamin D_3_ concentration in 5 women in Spain, was reportedly 16 nmol/L (37), considerably higher than other reports. Another study reported HM 25(OH)D_3_ concentrations in the nanomolar range (55) more typically associated with serum and plasma and inconsistent with other reports described in this review. Wang et al. reported mean 25(OH)D_3_ values from two groups of women of 4.3 and 9.9 nmol/L (56).

In unsupplemented women, comparison of 25(OH)D_3_ and vitamin D_3_ mean or median values suggests, either similar concentrations (31, 41, 53) or marginally higher 25(OH)D_3_ concentration (38, 41, 42), with the exception of one study that reported higher vitamin D_3_ (37). However, the upper values for vitamin D_3_ concentrations can be ~10 times higher than for 25(OH)D_3_ (36, 38, 41, 53), depending on the population. As a result, 25(OH)D_3_ concentration tends to be less variable between populations (not detected to 1.13 nmol/L) compared with vitamin D_3_ (0.02–13.37 nmol/L) (53). Studies where vitamin D_3_ supply is regular or high, either from supplementation or UVB exposure, tend to report higher vitamin D_3_ concentrations. Stoutjesdijk et al. measured vitamin D metabolites in a number of different populations in different continents and found variable ratios of 25(OH)D_3_ and vitamin D_3_. The highest concentrations of vitamin D_3_ were found in rural women from Tanzania with likely year-round UVB exposure. However, 25(OH)D_3_ concentrations in Tanzanian women were similar or slightly higher than the other populations. While the populations in Vietnam had generally similar or slightly higher vitamin D_3_ concentrations, the upper limit of the range for vitamin D_3_ was considerably higher than for 25(OH)D_3_. In the Netherlands, mean 25(OH)D_3_ concentrations were double that of vitamin D_3_.

## 5. Relationship between maternal and infant vitamin D status and response to supplementation

Higher maternal serum 25(OH)D_3_ concentration was associated with higher HM 25(OH)D_3_, vitamin D_3_ and ARA in observational studies (42, 53) and randomized controlled trials (34, 38, 39). In addition, in randomized controlled trials, maternal serum vitamin D_3_ was significantly positively associated with HM vitamin D_3_ and/or ARA (34, 39). In a maternal supplementation study (38), the significant correlation observed between serum 25(OH)D_3_ at 36 weeks gestation and HM concentration at 2 weeks postpartum was not evident at 2 months postpartum. Median HM 25(OH)D_3_ concentration was reportedly ~0.5–2% of 25(OH)D_3_ serum concentrations in observational studies (42, 53).

Maternal vitamin D_3_ supplementation in pregnancy (39) and during lactation (34, 35) increased HM vitamin D content. A fourth study also showed an effect of different dosages of vitamin D_3_ given in the third trimester, but the effect and difference from the placebo was only evident when outliers were removed (38). The vitamin D_3_ dosages in the studies ranged from 400 to 6,000 IU/d (10–150 μg/d) (35, 38, 39) or as a bolus dose (150,000 IU) (34). The studies reported no or only moderate increases in 25(OH)D_3_ concentration and larger increases in vitamin D_3_ concentration in HM (Table 3). Wall et al., provided maternal vitamin D_3_ up to 2,000 IU/d between 27 weeks gestational age (GA) and delivery, and while maternal vitamin D status and HM vitamin D content were increased, the median (range) ARA in milk remained low at 56 IU/L (21–148) (38). Stoutjesdijk et al. supplemented mothers with between 10 and 85 μg/d (400–3,400 IU) from 20 weeks GA until 4 weeks postpartum. Milk ARA increased in a dose-dependent manner with a median in the highest-dose group at 4 weeks postpartum of 156 IU/L (39). Dawodu et al. gave maternal doses of either 600 IU or 6,000 IU vitamin D_3_ daily for up to 7 months from 1 month postpartum. While there was a marginal increase in milk ARA with the 600 IU dose (8.1 to 14.3 IU/L), 6,000 IU/d increased milk ARA from 8 to 144 IU/L (35). Oberhelman et al. supplemented women for 28 days during lactation with 5,000 IU. Milk vitamin D_3_ concentration increased and largely reached a plateau after 3 days, with mean vitamin D_3_ of around 20 nmol/L (34). Two studies had data to show that maternal vitamin D supplementation improved HM vitamin D and infant status (34, 35). The study of Oberhelman et al. increased mean 25(OH)D_3_ concentration in infants from 42 to 98 nmol/L (34). Dawodu et al. raised infant 25(OH)D_3_ concentration from 32 nmol/L to 92 nmol/L with maternal supplementation of 6,000 IU, increasing the percentage of infants with 25(OH)D_3_ concentration above 50 nmol/L from 19 to 89%, and this regimen was comparable to infant supplementation with 600 IU/d (35). Two studies (34, 42), with serum samples also from infants, did not show any relationship between infant vitamin D status (25(OH)D_3_ concentration) and measures of maternal or HM vitamin D content.

## 6. Discussion

In this review, we had the twin aims of collating, comparing and contrasting published data on HM vitamin D concentrations measured by LC–MS/MS, and reviewing these data in the context and limitations of the reported analytical methods. The reviewed manuscripts were a mixture of method development reports and human studies, providing data from convenience, observational and vitamin D supplementation trials.

### 6.1. Pre-analytical factors: sample collection protocols and macronutrient content

HM sampling protocols may be an important factor contributing to reported HM vitamin D content. Lipid type and content of HM is known to vary between women, and within and between feeds (13, 57) and may influence vitamin D content (42). Diurnal (58) and seasonal factors (42) also need to be considered since seasonal variation in UVB availability will affect vitamin D status and could confound patterns of vitamin D concentrations observed during gestation and lactation (59). A recent meta-analysis also suggested an association with season and infant age (30). As optimal protocols for HM collection are not established for micronutrient HM content (13, 17) it is essential to report on the protocol for HM collections. Such information, together with data on stage of lactation and milk volume, will aide in the interpretation of HM composition data. Meanwhile, new methods such as dried milk spot sampling, are being investigated (13).

### 6.2. Analytical aspects: reporting

The studies identified in this review demonstrate inconsistency in reporting analytical methods. Partly this reflects the range of types of publication, from very detailed methodological reports, to observational studies or randomized controlled trials. While a number of reports provided sufficient detail to replicate methods and provided data on method validation, others reported minimal method details and/or referenced primary method papers which focussed on serum rather than HM. Such shortcomings in method descriptions makes replication of, or advancements in, methods difficult, if not impossible, at best hindering progress and at worse reporting unreliable data. In a recent example, albeit a HPLC method with UV detection, scant method details are provided, there is no analytical method information and it is not clear whether 25(OH)D or vitamin D, or both were measured (22). Unless the full assay has been published previously for the same matrix, in this case HM, full details of the extraction procedure are required, including starting volume of HM and volumes of chemicals used in the extraction procedures. Chromatography and mass spectrometric parameters should also be included.

While it may be unrealistic to expect a full validation of all methods to a clinical or internationally recognized standard, reporting of certain basic data are required to assess the quality and reliability of the data. This includes LOD/LOQ and a description of how values below these limits are handled; such considerations are frequently under-appreciated and often not discussed in nutritional biomarker literature (60). In addition, assay precision, extraction recovery and how they were derived should be detailed. This is important because in some methods, although isotopically labeled standards were used to correct for recovery, these were not always included from the start of extraction. The ability to include online supplementary material negates word count restrictions and allows the inclusion of full details of analytical methods and method validation parameters. Reporting of data, as discussed by others (17), needs to include actual numbers with mean or median and a measure of the variance, including the range; graphical representation alone is not sufficient. As above, the inclusion of online supplementary material allows summary data in to be included without duplication of results in the main manuscript. Overall, absence of analytical method detail means that study quality cannot be assessed, and consequently data may be excluded from collation for reviews and policy making decisions (17).

### 6.3. Analytical aspects: sensitivity

Analytical sensitivity is an important challenge for HM analyses because of the observed low concentrations of vitamin D metabolites. This review highlights a wide-range of LOQ, from <1 pmol/L to 18 nmol/L. The advantage of the lower LOQs is likely increased precision and more reliable quantitation at the lower concentrations. In methods with poor sensitivity, many values can be near or below the reported limits. In some cases, reported concentration ranges include values below the LOQ but the precision of these values is not indicated. This is particularly problematic where the LOQ is high, e.g., Oberhelman et al. report an LOQ of 18.2 nmol/L and LOD of 2.5 nmol/L (34). While higher LOQs raise the question on the usefulness and reliability of the method, the requirement of very low values may also be unnecessary, or at least, not physiologically relevant. If the vitamin D_3_ concentration of HM was 1 nmol/L (0.38 ng/mL) then 800 mL of HM would provide 0.31 μg/d of vitamin D_3_, 30-times less than current US recommendations for infants (400 IU/d (10 μg/d)) (61). The question also arises of how to handle data below the LOQ, particularly when the LOQ is high. Approaches for handling values below the LOD/LOQ include excluding values, use of a surrogate / imputed value (e.g., half the LOD/LOQ, LOD/sqrt (2) (59)) and multiple imputation. Exclusion of data is not recommended since non-detectable values may provide as much information as concentrations that can be measured.

### 6.4. Analytical aspects: accuracy

A limitation of all the HM vitamin D assays is the ability to establish accuracy. Over the last two decades, considerable effort has been made to improve the accuracy of serum assays for 25(OH)D. This has involved the development of reference measurement procedures (RMP) and certified reference materials (e.g., NIST Standard Reference Materials), as well as external quality assurance schemes (e.g., Vitamin D External Quality Assurance Scheme, DEQAS), and was only possible with considerable time and resources, such as can be afforded to a biomarker of such importance for clinical and research use. Similar resources are unlikely to be available for HM vitamin D analysis, thus the true value will be difficult to establish. Certified reference materials are available for formula milk with assigned vitamin D_2/3_ and/or 25(OH)D_3_ values (Table 4) and may be used to establish some degree of confidence in an assay. However, these preparations are unlikely to be representative of HM. Similarly, while accuracy of chemical solutions can be demonstrated, this is unlikely to be representative of accuracy in the biological matrix. The standard addition method may be used to estimate accuracy, but similarly, if the added standard does not behave in the same way as endogenous compound (due to for example, location within lipoproteins, protein binding etc.) then this approach will not provide a true measure. This demonstrates the need to provide as much information as possible about a method, including precision or repeatability, in order to provide confidence in the chosen approach. In house quality control materials to monitor assay performance over time are likely required. Despite the relative ease of HM collection and less restriction on volume than for example, whole blood or serum, commercial HM is not readily available and may be expensive. Access to HM from breast milk banks is possible but can require an extensive ethical approval process, which may not be feasible for method development or quality control material purposes.

**Table 4:** Reference materials for dried milk.

Product	Product number	Vitamin D forms	Origin of values
Infant/Adult Nutritional Formula II (milk/whey/soy-based)	SRM 1869	Vitamin D_2_ Vitamin D_3_	Reference values (manufacturer and NIST-collaborating laboratories)
Infant Nutritional Formula (milk-based)	NIST RM 8260	Vitamin D_3_	Provided by manufacturer
Adult Nutritional Formula (high protein)	NIST RM 8261	Vitamin D_2_ Vitamin D_3_	Provided by manufacturer
Whole milk powder	SRM 1549a	25(OH)D_3_ Vitamin D_3_	NIST
Whole milk powder	ERM-BD600	Vitamin D_3_	Informative value due to wide variation between participating laboratories

ERM, European Reference Material; NIST, National Institute of Standards and Technology; RM, reference material; SRM, Standard Reference Material.

In future, researchers should explore the sharing of matrix-matched, internal quality control materials to explore agreement between methods. In this incremental fashion, some consensus may be reached on true value for a given sample and, in the absence of reference method procedures and other high-level approaches, go some way to standardizing measurement, or at least provide a better understanding of the bias between methods.

### 6.5. Vitamin D concentrations in human milk

The data presented here suggest a potential wide range of vitamin D concentrations in HM. In the majority of studies, 25(OH)D_3_ and vitamin D_3_ concentrations were in the low nanomolar range (<10 nmol/L), similar to concentrations reported using earlier methods (62). However, some studies suggested considerably higher 25(OH)D_3_ (55) and vitamin D_2_/_3_ concentrations (37). Such values appear inconsistent with the majority of studies and may be due to short-comings in analytical methods. Two other recent studies have described similarly high concentrations. One study utilized a HPLC method and reported the unlikely situation where the 2(OH)D_3_ concentration was 30 nmol/L higher in HM than in serum in both vitamin D-supplemented and unsupplemented mothers (63). The second study used an automated chemiluminescence analyzer validated for serum but not for HM and described high analytical limits (18 nmol/L) (64, 65). These assays may have suffered from inadequate specificity and cross-reactivity leading to spurious elevated concentrations.

Conversely, a number of other studies reported unmeasurable, zero concentration (46), or unquantifiable vitamin D metabolite concentrations (34) mostly likely due to poor sensitivity, and/or poor extraction recovery. These large differences in concentrations may be due to the many challenges of measuring vitamin D in HM rather than true biological differences. Furthermore, these studies demonstrate that methods for the extraction and analysis of vitamin D metabolites from serum or plasma are unlikely to be suitable when directly applied to the different matrix of HM. Whereas inter-individual differences in fasted serum or plasma composition are relatively small, HM can be highly heterogeneous in respect of fat and protein content and have a different composition across a feed, a day, and across time, that requires a robust extraction methodology to reliably and consistently extract vitamin D metabolites.

The relative importance of different forms of vitamin D in HM is not certain. Not all studies measured both forms, and 25(OH)D_3_ and vitamin D_3_ are generally not correlated (41). Vitamin D_2_ forms do not make a meaningful contribution to the vitamin D content of HM except through significant maternal vitamin D_2_ supplement intake. Although an early study indicated 25(OH)D_3_ concentrations were 8-fold higher than vitamin D_3_ concentrations (23) a subsequent study demonstrated greater vitamin D_2_ or vitamin D_3_ concentrations than 25(OH)D_2_ or 25(OH)D_3_ concentrations in HM after supplementation and/or UV exposure. The exception was for the low dose (10 μg/d vitamin D_3_) participants where 25(OH)D_3_ was more than two-times greater than vitamin D_3_ (52). In a case study of a women taking 100,000 IU of vitamin D_2_ daily during pregnancy, which led to maternal vitamin D_2_ and 25(OH)D_2_ serum concentrations of more than 1,300 nmol/L, vitamin D_2_ concentrations in HM were over 300 nmol/L, but 25(OH)D_2_ concentration was around 20 nmol/L (66). Other studies have stated vitamin D is a greater contributor to ARA than 25(OH)D although metabolite concentration data are not reported (67, 68). Thus, it appears, at least when there is large single bolus dose or regular supply of vitamin D (from supplements or generated through cutaneous synthesis after UV exposure), vitamin D rather than 25(OH)D becomes the major vitamin D form in HM. Consequently, there is much greater variability in vitamin D concentration than 25(OH)D concentration in HM. Evidence from the studies reviewed here are consistent with this notion as there is greater variance in vitamin D_3_ concentration than 25(OH)D_3_ concentration due to UVB exposure (53) and maternal vitamin D supplementation (38, 39). Conversely, a recent meta-analysis concluded that 25(OH)D is the major contributor to HM vitamin D content (30). However, the meta-analysis included all studies with measured concentrations of 25(OH)D and/or vitamin D without consideration of the reliability of different methods. The study also reported a significant difference in 25(OH)D concentration between analytical methods (LC–MS/MS, HPLC with competitive binding assay, or “Other”) with the “Other” group reporting ~10 times higher 25(OH)D concentration than chromatography-based methods (30), potentially skewing results toward 25(OH)D. Analytical factors related to recovery during extraction, matrix effects and sensitivity may also affect interpretation when comparing between studies. Overall, under conditions of low vitamin D supply, either or both forms can contribute to HM vitamin D content as demonstrated by the studies reviewed here (41, 43). The higher biopotency of 25(OH)D suggests that both forms should be measured in HM. Vitamin D may be more important than 25(OH)D where supply is increased through UVB exposure and/or supplementation. The importance of the parent form of vitamin D to HM total vitamin D content may be related to relatively lower binding affinity vitamin D compared with 25(OH)D. As a result, there is a greater level of free circulating vitamin D that may diffuse across cell membranes into HM. This is in contrast to 25(OH)D that predominantly circulates bound to DBP and that relies on this binding for cellular uptake through the megalin-cubulin pathway (69, 70).

The manuscripts reviewed here show that vitamin D supplementation during pregnancy or lactation can improve the vitamin D content of HM (34, 35, 38, 39), and infant vitamin D status (34, 35). In an earlier study, Wagner et al. provided mothers with either 400 or 6,400 IU/d from 1 month post-partum. Milk ARA did not increase in the 400 IU/d group, but rose steadily in the 6,400 IU to reach a mean ARA of 874 IU/L after 6 months, and mean infant 25(OH)D_3_ concentration of 115 nmol/L, a value very similar to that achieved with infant supplementation of 300 IU/d (108 nmol/L) in the same study (67). Similar findings were subsequently reported from a larger randomized controlled trial with maternal doses of 6,400 IU in the USA, whereas the same study found that maternal doses of 2,400 IU did not support infant vitamin D status (62). A recent meta-analysis of maternal vitamin D supplementation and its effect on milk vitamin D content and infant vitamin D status reported a significant increase in milk ARA with vitamin D supplementation and a prediction equation of “ARA = 0.099 × vitamin D dose (IU/D) -30” (71). In addition, the authors calculated that for each 1,000 IU of maternal vitamin D supplementation, infant 25(OH)D concentration increased by 6.8 nmol/L (71). These data suggest that lower doses of maternal vitamin D supplementation are unlikely to provide a milk vitamin D content sufficient to meet a vitamin D intake of 400 IU/d (72, 73). However, it is also uncertain whether the higher doses used in some studies are necessary and thus the maternal supplementation dose required to maintain adequate infant vitamin D status is yet to be confirmed (71, 74, 75).

### 6.6. Other vitamin D forms

This review has focussed on 25(OH)D and ergocalciferol (vitamin D_2_) and cholecalciferol (vitamin D_3_) however, other vitamin D metabolites may also potentially contribute to total vitamin D content. Early reports suggested vitamin D sulphate, a water-soluble conjugate of vitamin D, may be a significant source of vitamin D (76, 77). Although considered biologically inactive (58), hydrolysis of the conjugated form may liberate biologically active metabolites (32). However, non-specific methods and instability of the sulphated form during saponification have been identified as limitations to these methods and subsequent reports with greater specificity suggest the sulphated form is not a major contributor to the vitamin D content of HM (78–81). More recently, a LC–MS/MS method that includes sulphated forms has been developed (32) with mean values for sulphated forms between 87 and 261 pmol/L, comparable to lipid-soluble vitamin D metabolites, but no other data have since been published.

Metabolites downstream of 25(OH)D (1,25(OH)_2_D and 24,25(OH)_2_D) are unlikely to meaningfully contribute to total milk vitamin D content since in serum, these metabolites circulate at around 1 and 10% of 25(OH)D concentration, respectively. Data from cows milk, even after supplementation, suggest none at all, or insignificant concentrations of 1,25(OH)_2_D and 24,25(OH)_2_D. The LC–MS/MS method of Gomes et al. included 1,25(OH)_2_D_3_ and 24,25(OH)_2_D_3_ and mean concentrations in human milk samples used in the validation of their method and with concentrations greater than the LOQ, were 1.14 and 7.68 pmol/L, respectively (31). Hollis earlier reported concentrations of 13, 135 and 83 pmol/L for 1,25(OH)_2_D_3_, 24,25(OH)_2_D_3_ and 25,26(OH)_2_D_3_, respectively, although these were in the context of relatively high milk 25(OH)D_3_ of 780 pmol/L (23). In other reports, 1,25(OH)_2_D_3_ and 24,25(OH)_2_D_3_ were not detected (82, 83). Consistent with the data reviewed in this manuscript for 25(OH)D and vitamin D, the limited data available for dihydroxy metabolite concentrations in milk are not consistent, likely due to small sample sizes, different populations with varying vitamin D status, and different analytical methods with varying degrees of specificity and sensitivity. A further compound of potential relevance to the vitamin D content of HM is the C3-epimer of 25(OH)D (3-epi-25(OH)D), an isomer of 25(OH)D. Although the biological relevance of the 3-epi-25(OH)D remains unclear, if it is not chromatographically separated from 25(OH)D then estimates of vitamin D status in serum can be inflated. In particular, it is known that the 3-epi-25(OH)D is generally higher in neonates than other age groups peaking at up to 3 months of age, before declining, and can significantly contribute up to infant status (84–86). The origin of high 3-epi-25(OH)D in neonates is not certain, but appears unlikely to be from the maternal circulation as maternal concentrations are generally similar or lower than those observed in infants and evidence suggests the 3-epi-25(OH)D does not readily cross the placenta (87). While a maternal contribution through lactation cannot be ruled out, particularly where 3-epi-25(OH)D may be increased due to vitamin D supplementation, current thinking is that 3-epi-25(OH)D compounds are a product of immature vitamin D infant metabolism in early life (86, 87). There is little evidence to support appreciable amounts of 3-epi-25(OH)D in HM. Of the studies reviewed here, four stated that 3-epi-25(OH)D was chromatographically resolved (31, 34, 36, 55), and of these, two included validation of 3-epi-25(OH)D quantitation in HM (31, 55), although one presented no concentration data (31). The study that reported disparate values for 25(OH)D_3_ concentration in HM, also reported extremely high 3-epi-25(OH)D_3_ concentrations in HM, equal to around one-third of the HM 25(OH)D_3_ concentration, although maternal plasma 3-epi-25(OH)D_3_ was below the LOQ (55).

### 6.7. Future directions

As highlighted by the range and often multi-stepped procedures for vitamin D measurement in HM, the suitability of a method designed for serum 25(OH)D measurement is questionable. The unique matrix of HM, with high lipophilic compound content, as well as the relevance of both 25(OH)D and vitamin D compounds with differing polarities, means that methods specifically designed for milk are required. The analysis of vitamin D in HM remains challenging, in particular the essential but laborious extraction techniques. Protein precipitation, liquid–liquid extraction and/or saponification remain the major extraction techniques to separate vitamin D from the main other lipophilic compounds in HM. In future, advances in solid phase extraction (SPE) and supported-liquid extraction may provide simplified approaches to purification of vitamin D metabolites (25). Developments in chromatography, including supercritical fluid chromatography, as used by Oberson et al., to measure vitamin D metabolites in HM, purportedly can provide a shorter run time and better chromatographic separation than liquid chromatography (36). The apparent low endogenous concentrations of vitamin D metabolites in HM and/or poor extraction efficiency, often leads to the requirement for large sample volumes for extraction. As discussed above, chemical derivitisation can improve sensitivity and ionization efficiency but only two types have reportedly be used for vitamin D detection in HM, whereas a larger number of derivitisation reagents have been applied to vitamin D metabolites in serum (25). Finally, further improvements in vitamin D analysis may be obtained from developments in mass spectrometer sensitivity and specificity, such as provided by high resolution techniques, in particular hyphenated to liquid chromatography.

## 7. Conclusion

The studies reviewed here have been performed across a number of continents however, they were generally small in size, with around 20 up to 100 participants. Studies report a wide-range of concentrations of both 25(OH)D and vitamin D, the latter modifiable with vitamin D supplementation. In general, vitamin D concentrations in the milk of women who are not taking vitamin D supplements and not exposed to regular UVB are low and unlikely, alone, to supply infants with sufficient vitamin D to maintain infant vitamin D status. In studies of milk vitamin D concentration, total milk volumes are rarely reported and limits the ability to determine accurately total vitamin D intake in exclusively breastfed infants. This makes determining any consensus or public health policy, challenging. The ongoing Mothers, Infant, and Lactation Quality (MILQ) Study was established to obtain globally representative data on the micronutrient content of HM with the aim to establish reference ranges for a range of vitamins and minerals (16). The study was implemented in Brazil, Denmark, The Gambia and Bangladesh, and will help to plug some of the gaps in knowledge around the micronutrient content of HM, including vitamin D.

Application of LC–MS/MS has significant potential to contribute to the generation of data on HM vitamin D metabolite concentrations. No doubt the analysis of vitamin D in HM is challenging and pre-analytical, analytical and post-analytical factors may influence the reported outcomes and should be considered when interpreting the concentration data. While a number of methods have been published, few studies have reported data from more than a small number of participants, partly due to the large volume of HM required for certain assays. Furthermore, differences in collection protocols and, in particular, uncertainty over the quality/validity of the analytical methods, including measurement of specific vitamin D forms, means that it is difficult to say with confidence typical vitamin D concentrations in HM. Future work should focus on improving current analytical methods and their validation, on novel methods to improve extraction and analysis of vitamin D metabolites, and on larger studies to characterize HM milk vitamin D metabolite concentrations, in particular in relation to maternal and infant vitamin D status.

## Author contributions

KJ, SM, and AK conceived the review. KJ and SM compiled and interpreted the data. KJ wrote the first draft of the manuscript. All authors contributed to manuscript revision, read, and approved the submitted version.
