# Supplementary material for: Quantification and reporting of vitamin D concentrations measured in human milk by LC–MS/MS
Source: Front Nutr. 2023 Nov 16;10:1229445. doi: 10.3389/fnut.2023.1229445 (PMC10687194; doi:10.3389/fnut.2023.1229445)
Supplement: Supplementary file 1 [file Table_1.DOCX]

**Quantification and reporting of vitamin D concentrations measured in human milk by LC-MS/MS**

**Kerry S Jones, Sarah R Meadows, Albert Koulman**

Nutritional Biomarker Laboratory, MRC Epidemiology Unit, University of Cambridge, Cambridge, UK

**Supplementary table**

Supplementary Table 1. Human milk collection protocols^1^

| **Study** | **Breast milk collection protocol** |
| --- | --- |
| Kamao, 2007 (41) | “Approximately 50 mL of human breast milk was collected by manual expression at an intermediate time during suckling.” |
| Oberhelman, 2013 (34) | “Women collected their breast milk via a breast pump or self-expression.” “Because measurements  of vitamin D metabolites do not differ between whole milk and milk whey, no attempt was made to distinguish foremilk and hindmilk collection.” |
| Jan Mohamed, 2014 (45) | “Breast milk samples were collected using a mini electric breast pump.” |
| Vio Streym, 2016 (42) | “…foremilk (i.e., milk before feeding the child) and hindmilk (i.e., milk after feeding the child) were manually collected by the mothers following directions in handed-out instructions.” |
| Wall, 2016 (38) | Standard protocol was followed ^a^ “Samples were collected either manually or with the use of a human-milk pump. Breast milk was collected directly into a small sampling bottle that was provided to the mothers. If a pump was used, milk was collected into a clean container that came with the pump and was poured into the sampling bottle. Breasts and hands were cleaned, but soap and lotions were avoided because they may have contained chemicals that could interfere with the measurement of breast-milk VDA. The sample was collected at the regular feeding time in the morning, 2 h after the previous breastfeed (this method was used to allow for some consistency between samples). A full breast was expressed to ensure that the hindmilk was collected.” |
| Stoutjesdijk, 2017 (46) | “The mothers in the Netherlands, Curacao and Vietnam were instructed to save a 25 ml of milk sample that was taken from a completely emptied breast around noon. A quantity of 25 ml milk samples in Malaysia were collected in the morning as far as possible. Tanzanian mothers collected a midstream sample at an undefined time during the day.” |
| Dawodu 2019 (35) | “A 25 ml aliquot of full breast milk expression was collected at  visits 1, 4 and 7 and frozen.” |
| Stoutjesdijk, 2019 (39) | “The participants were instructed to collect the full amount of breast milk from a single breast around noon (10·00–14·00 hours) on the day before, or on the day of, blood sampling, using a standardised protocol. The milk was collected either manually or using a breast milk pump. To ensure homogenisation, they were carefully swerved and subsequently transferred to two sampling tubes. The milk samples were stored in the participants’ freezer.” |
| Wang, 2019 (48) | “The mothers were required to collect the BM by hand expression after the morning feed between 8 and 11 am. Fresh milk samples were transported to the laboratory on ice and stored at −80°C prior to analysis” |
| Gjerde, 2020 (47) | Collected at beginning, middle and end of a chosen feed and pooled for analysis |
| Oberson, 2020 (36) | Not reported. Commercial breast milk samples from healthy single donors (Lee Biosolutions, Maryland Heights, USA) |
| Tsugawa, 2021 (43) | “Midmilk (1989) and mid-hindmilk (2016-2017) were manually collected by the mothers following directions in handed-out instructions” |

^1^ Reference details for each study can be found in the main manuscript
